# Constructing an automatic diagnosis and severity-classification model for acromegaly using facial photographs by deep learning

**DOI:** 10.1186/s13045-020-00925-y

**Published:** 2020-07-03

**Authors:** Yanguo Kong, Xiangyi Kong, Cheng He, Changsong Liu, Liting Wang, Lijuan Su, Jun Gao, Qi Guo, Ran Cheng

**Affiliations:** 1 Department of Neurosurgery, Peking Union Medical College Hospital, Chinese Academy of Medical Sciences, NO.1 Shuaifuyuan Hutong of Dongcheng District, Beijing, 100730 China; 2 Department of Breast Surgical Oncology, National Cancer Center/National Clinical Research Center for Cancer/Cancer Hospital, Chinese Academy of Medical Sciences and Peking Union Medical College, Beijing, 100021 China; 3 Guangdong Provincial Key Laboratory of Brain-inspired Intelligent Computation, Department of Computer Science and Engineering, Southern University of Science and Technology, Shenzhen, 518055 China; 4 Department of Electronic Engineering, Tsinghua University, Beijing, 100084 China; 5 Shenzhen JOY SMART Lab INC, Shenzhen, China; 6 School of Media and Health Communication, Shenzhen University, Shenzhen, China; 7 Cardiovascular Epidemiology Unit, Department of Public Health and Primary Care, University of Cambridge, Cambridge, UK

**Keywords:** Severity-classification model, Acromegaly, Facial photographs, Deep learning

## Abstract

Due to acromegaly’s insidious onset and slow progression, its diagnosis is usually delayed, thus causing severe complications and treatment difficulty. A convenient screening method is imperative. Based on our previous work, we herein developed a new automatic diagnosis and severity-classification model for acromegaly using facial photographs by deep learning on the data of 2148 photographs at different severity levels. Each photograph was given a score reflecting its severity (range 1~3). Our developed model achieved a prediction accuracy of 90.7% on the internal test dataset and outperformed the performance of ten junior internal medicine physicians (89.0%). The prospect of applying this model to real clinical practices is promising due to its potential health economic benefits.

To the Editor,

Acromegaly is generally caused by the persistent excessive secretion of the growth hormone (GH), usually resulting from pituitary adenomas. Due to its insidious onset, vague symptoms, and slow progression, the diagnosis of this disease is usually delayed rendering severe complications like cardiovascular diseases and treatment difficulty. A convenient screening method is imperative. The typical facial features of acromegaly are critical clues for the preliminary diagnosis, including enlarged nose and brow, prominent jaw and zygomatic arch, thick tips, and swelling facial soft tissue. In our previous study, we developed an algorithm model for the automatic detection of acromegaly from facial photographs using machine learning, whose positive predictive value (PPV) achieved 96%, outperforming the neuroendocrinologists [1]. However, this study failed to differentiate the severities and stages of the acromegaly. In our present study, we aimed to train more facial photographs of patients with acromegaly at different severity levels to construct an updated algorithm model with the function of both automatic diagnosis and severity-classification. The materials, methods, and results are shown in detail in the Additional file 1.

We totally included 716 subjects (339 women, 54.3 ± 12.7-year-old; 377 men, 52.7 ± 10.1-year-old), contributing to a total of 2148 photographs at different severity levels (1911 in the training dataset and 237 in the test dataset). By visual inspections, twenty board-certified neuroendocrinologists separately gave each photograph a score reflecting its severity [normal or very slight (score 1), mild or moderate (score 2), and severe (score 3)]. The modal numbers were chosen as the final scores (for all photographs, the proportions of the occurrence of the modal numbers were over 80% among the 20 scores). We calculated a Spearman rank-order correlation coefficient with *p* values to validate the significant positive correlation between the score and the real clinical severity, reflected by the tumor size, tumor maximum diameter, serum GH level, serum insulin-like growth factor (IGF)-1 level, and ki67% (all *P* < 0.01; Table S1, Figure S1), which could be seen as the gold standard used to assign the true severity label to each patient.

The Face Recognition Library was used to do the face detection. Specifically, we first used OpenCV Cascade Classifier with a Haar Cascade to detect the face in a color image and get the face bounding rectangle box. In order to retain the forehead and chin information, we then increased the height of the bounding box by expanding both the top and bottom. At last, we cropped and resized all the detected bounding boxes to the same pixel dimensions of 160 × 160 pixels (Figure S2). We augmented the existing data by changing the brightness, changing the saturation, adding Gaussian noise, and flipping horizontally (Figure S3). Face frontalization was achieved using the method proposed by Sagonas et al. [2]. The architecture of our model is shown in Fig. 1. We used the pre-trained Inception ResNet V1 as our feature extractor for face recognition based on CASIA-WebFace dataset [3]. At the end of our model, there were two branches, one for the softmax loss and the other for the center loss. In the softmax loss branch, instead of using fully connected layers, we proposed to use a 1 × 1 convolutional layer and a global average pooling layer as the final classifier, which could reduce the number of parameters and possibility of overfitting. Then, we used the cross-entropy as our softmax loss function [4]. In the center loss branch, we used a fully connected layer to produce a 512-dimensional vector as the learned features. To take advantage of the discriminative power of the features, we created three trainable 512-dimensional vectors corresponding to three classes respectively. Then, we used the distance between the vectors and the features as our center loss, i.e., the input images belonged to the same class should have the same features.

**Fig. 1:**
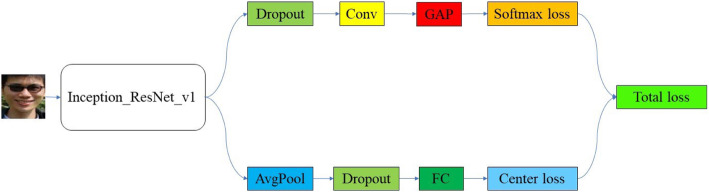
The architecture of our proposed model. Conv represents the 1 × 1 convolutional layer; the GAP, AvgPool, and FC are the global average pooling layer, the average pooling layer, and the fully connected layer, respectively. In this work, the rate of dropout was set to 0.8, the activation function of the convolution layer is ReLU, and there is no activation function in the fully connected layer

The trained model was tested by a separate test dataset, which was labeled by the same neuroendocrinologists. The test data were not seen before and collected from different hospitals, containing a total of 79 subjects with 237 photographs, among which, 43 photographs were scored 1, 93 photographs scored 2, and 101 photographs scored 3. The total prediction accuracy of our proposed model was 90.7%, where 22 photographs had the incorrectly predicted scores. For score 1 class, our model had a precision of 94.1%, a recall of 74.4%, and a F1-Measure of 0.831 (Table 1 (A)). To be more specific, the false-negative rate (FNR) was 1.03%, where two patients with acromegaly were predicted to be health (predicted to score 1 participants) over 194 participants with acromegaly (score 2 and score 3 participants). We also tested its performance against ten junior internal medicine physicians. The total prediction accuracy of our model was higher than that of the ten physicians (90.7% vs. 89.0%). For score 1 class, the physicians had a precision of 91.7%, a recall of 76.7%, and a F1-Measure of 0.835 (Table 1 (B)).

**Table 1:** Confusion matrix to evaluate accuracy, precision, and recall of the algorithm model

Predicted severity	Actual numbers in the test dataset			Total
	Score 1	Score 2	Score 3	
**A** Accuracy, precision, and recall of our algorithm model				
Score 1	32	1	1	34
Score 2	4	85	2	91
Score 3	7	7	98	112
Total	43	93	101	237
Precision	94.1%	93.4%	87.5%	
Recall	74.4%	91.4%	97.0%	
F1-Measure	0.831	0.924	0.920	
Total prediction accuracy	-	-	-	90.7%
**B** Accuracy, Precision, and Recall of Physicians				
Score 1	33	3	0	36
Score 2	5	83	6	94
Score 3	5	7	95	107
Total	43	93	101	237
Precision	91.7%	88.3%	88.8%	
Recall	76.7%	89.3%	94.1%	
F1-Measure	0.835	0.888	0.914	
Total prediction accuracy	-	-	-	89.0%

Previously published related models, including our own developed one [1], could only tell whether or not the photography was an acromegaly. To our knowledge, the present model was the first one with the function of severity-classification, thus had some breakthrough implications. Of note, the accuracy 90.7% was lower than that of our previous study (PPV 96%) [1]. This was because the outcome of the severity-classification model was a polytomous variable rather than a dichotomous variable and had to require much more potential input characteristic variables. This study harbored several limitations. Firstly, because the acromegaly itself is an uncommon disease, the training data size was not large enough. To achieve a higher accuracy, we have been focusing our efforts on two main directions: (1) accumulating more photography to enlarge the data size and (2) introducing the face classification with 3D information of video frames, which would overcome those difficulties in image pre-processing and increase the accuracy for acromegaly screening. Secondly, although the test dataset we used were collected from different hospitals, the data size was small. We will test its generalization on a greater scale. Thirdly, the study was performed mainly in Asian population and may be limited to be extrapolated to other populations. We have been trying to estimate its applicability to Caucasian and Black populations and anticipate completing this work by the end of this year. Nevertheless, the grassroots solution for the model generalization was still incorporating more training data of other populations to further optimizing the algorithm.

In conclusion, this developed model achieved a relatively high sensitivity and specificity for automatic diagnosis and severity-classification of acromegaly. Due to its usefulness in helping people conduct the self-screening conveniently, such as uploading one’s selfie to a specifically developed mobile min app, resulting in acromegaly’s early diagnosis and thus early treatment, our work could greatly save medical resources, improve medical efficiency, alleviate imbalances in health development between regions, and has significant health economic benefits in the world.

## Supplementary information

**Additional file 1..** The materials, methods, results and limitations of this study in detail.

**Additional file 2:.** Table S1. Spearman correlation coefficient results and p-values measuring the rank correlation.

**Additional file 3:.** Figure S1. The heatmap displaying the Spearman Correlation coefficient of the score and other features.

**Additional file 4:.** Figure S2. Face detection: the blue box represented the detected bounding box by the Face Recognition library. The red box represented the bounding box after we increased the height.

**Additional file 5:.** Figure S3. Examples of data augmentation methods, from left to right, we had the original image, the image with changed brightness, the image changed saturation, the image added Gaussian noise, the image flipped horizontally.

## Data Availability

All supporting data are included in the manuscript and supplemental files. Additional data are available upon reasonable request to corresponding author.

## Abbreviations

GH: Growth hormone
PPV: Positive predictive value
