# Supplementary material for: Constructing an automatic diagnosis and severity-classification model for acromegaly using facial photographs by deep learning
Source: J Hematol Oncol. 2020 Jul 3;13:88. doi: 10.1186/s13045-020-00925-y (PMC7333291; doi:10.1186/s13045-020-00925-y)
Supplement: Supplementary file 1 — Additional file 1.. The materials, methods, results and limitations of this study in detail. [file 13045_2020_925_MOESM1_ESM.doc]

**Additiona****l File 1**

**1 Methods and Algorithms**

**1.1 Datasets**

The study conforms to the Declaration of Helsinki [1]. All participants provided written informed consent. All patients whose pictures are shown here gave separate informed consent for showing their pictures. We recruited patients with acromegaly from several large general hospitals in China. The patients were required to provide their past photos at separate time points, i.e., 2 years ago, 5 years ago, 10 years ago, etc., which will reflect different stages of severity. Seventy healthy volunteers without acromegaly at these hospitals contributed some the aged-matched and sex-matched facial photographs.

In total, we included 716 subjects (339 women, 54.3 ± 12.7 years old; 377 men, 52.7 ± 10.1 years old). The acromegaly diagnosis was validated by non-suppressed GH in an oral glucose tolerance test and IGF-I levels per clinically used guidelines. These patients contributed a total number of 2148 of photographs at various time points. One test dataset was used for clinical validation, which did not overlap with the data used in the training dataset (a total of 637 subjects with 1911 photographs). The test dataset contains 79 subjects with 237 acromegaly photographs. All photographs were labeled with a score marked by twenty neuroendocrinologists (from Score-1 to Score-3).

**1.1.1 Clinical Laboratory Test Data**

Every patient’s disease course (months), tumor size (mm^3^), maximum tumor diameter (mm), serum growth hormone (GH) level, serum IGF-1 level, and Ki67% were collected. Through analysis, we aimed to prove that the scores given by the neuroendocrinologists are positively related to the clinical laboratory test data. We calculated a Spearman rank-order correlation coefficient and the p-value to test the non-correlation.

**1.1.2 Photograph Acquisition**

Since we planned to apply the final algorithm to the actual clinical setting, we did not set too many restrictions for the photo taking. The shooting angle was required within plus or minus 45 degrees centered on the standard coronal plane, and no specific limits were set to the shooting background. The cameras used for taking photos were neither limited, which actually included the Single Lens Reflex cameras, digital cameras, and mobile phone cameras, etc.

**1.2 Face Classification**

Prior to the facial classification, we grouped all acromegaly photos into groups of different severity by visual inspection [normal or very slight (Score 1), mild or moderate (Score 2), severe (Score 3)]. Twenty board-certified neuroendocrinologists together labeled these pictures. Then a modal score was assigned to each photograph. In case of discrepancy, more neuroendocrinologists were consulted to form a unified opinion. Generally, the scoring standards are described as follows:

**1.2.1 Score 1**: normal face, or there were signs of facial changes starting to happen, but it is very slight.

**1.2.2 Score 2**: not typical state, the acromegaly severity was mild (mainly including ala nasi, lips, zygomatic arch, and underjaw); or moderately typical state, which is the intermediate state between score 1 and score 3.

**1.2.3 Score 3**: typical state of acromegaly with severe facial sighs (the upper incisors are becoming spread apart severely, severe jaw prognathism, obvious frontal-bone enlargement, apparent nose enlargement, and the facial-features are becoming coarse).

**1.3 Method Outline**

To automatically identify acromegaly disorders using deep learning methods from facial images, we first detected the face bounding box in the facial photograph and then cropped and resized it to the same pixel dimensions. Next, we used a CNN model to automatically classify the face images according to the severity of acromegaly. Then we extracted the critical facial landmark locations. Finally, we used face frontalization methods to improve the diagnostic performance.

**1.4 Relationship** **Between Score and Severity**

We calculated a Spearman rank-order correlation coefficient and the p-value to test the non-correlation. Here, we calculated the two-sided p-value for the hypothesis test, which could indicate the probability of an uncorrelated system producing datasets that had a Spearman correlation at least as extreme as the one computed from these datasets.

**1.5 Image Pre-Processing**

**1.5.1 Face Detection**

To identify acromegaly disorders by using automated face recognition technology, we first needed to locate the face in the image. We used the Face Recognition library to do face detection. Face Recognition library was built on the basis of Dlib’s state-of-the-art face recognition based on deep learning model; the model has an accuracy of 99.38% on the labeled faces in the wild benchmark. Dlib is a modern C++ toolkit containing machine learning algorithms and tools for creating sophisticated software in C++ to solve real-world problems. It is used in both industry and academia in a wide range of domains including robotics, embedded devices, mobile phones, and large high-performance computing environments. Dlib's open source license allows us to use it in our application, free of charge. Herein, we first read a color image which included a face, and then used OpenCV Cascade Classifier with a Haar Cascade to detect the face and get the face bounding rectangle box. In order to retain the forehead and chin information, we increased the height of the bounding box, expanding top and bottom 1/5 height of the bounding box, respectively. At last we cropped and resized all the detected bounding boxes to the same pixel dimensions of 160*160 pixels, as shown in **Figure S2**.

**1.5.2 Data Augmentation**

Data augmentation means increasing the number of data samples. When you trained a deep learning model, what you are really doing is tuning its parameters such that it can map a particular input to some specific output. Our optimization goal was to identify the sweet spot with low model loss, and it can only be achieved when the parameters were well tuned. The parameter tuning of the model was based on the data that you feed it, the more the data, the better the performance of the model. However, data collection was subject to various costs, such as money, human effort, computational resources, and of course, time consumed in the process. Therefore, we had to augment existing data to compensate for the cost involved in further data collection. In this paper, we used several methods to augment our data, including changing the brightness, changing the saturation, adding Gaussian noise, flipping horizontally, and the results were shown in **Figure 1**.

**1.5.3** **Face Frontalization**

Frontalization is the process of reconstructing the frontal facing views which have been reported helpful for face landmark localization and face recognition [2]. In this study, we used the method proposed by Christos Sagonas et al. to produce frontalized views for improving the performance of facial landmark localization and acromegaly disorders diagnosis from facial images <https://paperpile.com/c/Ygowje/391H> [3]. First, we detected a face by using an off-the-shelf face detector and then cropped and resized the detected face to a standard coordinate system. Note that the facial feature samples were localized to align the photo with a reference face. Then we obtained the initial frontalized face by back projecting the appearance of the facial images to the reference coordinate system. Finally, we reconstructed the final frontalized face by borrowing the appearances from corresponding symmetric sides wherever facial features were poorly visible.

**1.6 Algorithm Development**

A convolutional neural network (CNN) contains an input layer as the network’s input, an output layer representing the multiple hidden layers and prediction results. The hidden layer typically contains pooling layers and convolutional layers. For the convolutional layer, a convolution operation is applied to the input and then the results are passed to the next layer as its input. The convolution emulates the response of an individual neuron to visual stimuli, where convolutional neurons process data only for their own receptive fields. The neuron does not connect to all the neurons in the next layer but only to a small region of it and all the neurons share these parameters for the connection. This character can not only preserve the spatial information of images but also reduce the parameters of the model. The pooling layer always follows the convolutional layers, and it combines the outputs of neurons at one layer into a single neuron for down-sampling, compresses and generalizes feature representations. There are two kinds of pooling layers, local pooling layers and global pooling layers.

CNNs are widely used in computer vision tasks. They have achieved desirable results in comparison with state-of-the-arts. In most of the available CNNs, the softmax loss function is used as the supervision signal to train the model as a classifier. However, it does not make full use of the discriminative power of the deeply learned features. In this paper, we used not only a softmax loss but also a center loss [4] to train the model.

$l=l_{s}+\lambda l_{c}$,

where $l_{s}$and $l_{c}$were the softmax and center loss function respectively and $\lambda\text{=}0.8$ was used for balancing the two loss functions.

The architecture of our model was shown in **Figure 2**. We used the pre-trained Inception ResNet V1 as our feature extractor for face recognition based on CASIA-WebFace dataset [5]. At the end of our model, there were two branches, one for the softmax loss and the other for the center loss. In the softmax loss branch, instead of using fully connected layers, we proposed to use a 1∗1 convolutional layer and a global average pooling layer as the final classifier, which could reduce the number of parameters and possibility of overfitting. Then we used the cross-entropy as our softmax loss function. In the center loss branch, we used a fully connected layer to produce a 512-dimensional vector as the learned features. To take advantage of the discriminative power of the features, we created three trainable 512-dimensional vectors corresponding to three classes respectively. Then we used the distance between the vectors and the features as our center loss, i.e., the input images belonged to the same class should have the same features.

**1.7 Algorithm Internal Testing and External Evaluation**

The algorithm evaluation process consisted of two main parts. The first part was internal testing, and the second part was external evaluation. The internal testing was based on the test dataset mentioned above. Specifically, the developed computer program would be used to recognize the face pictures in the test dataset, and a score from 1 to 3 should be given. Then we statistically analyzed the testing results, mainly including the accuracy, precision, and recall.

During the external evaluation phase, based on the same test dataset with scores marked by the 20 neuro-endocrinologists, we invited ten junior internal medicine physicians, who had already worked as a doctor in large and comprehensive first-grade hospitals in china for 3~4 years, to give a score according to the severity. Note that the research staff would inform the physicians of the scoring rules in advance and asked them to give the corresponding scores solely based on their own medical experience and overall impression. The mode was used to reflect the ten physicians’ overall scoring. We then compared the accuracy, precision, and recall between the developed computer algorithm and the doctors.

**2 Results**

**2.1 Photograph Collection and Classification**

In total, we collected 716 subjects (339 women, 54.3 ± 12.7-year-old; 377 men, 52.7 ± 10.1-year-old). These patients contributed a total of 2148 photographs at various time points. On average, every patient provides 3 photographs.

For the training dataset (a total of 637 subjects with 1911 photographs), after scoring, there were 191 subjects with 573 photographs scored 1 (97 women; 94 men), 239 patients with 717 photographs scored 2 (107 women; 132 men), and 207 patients with 621 photographs scored 3 (97 women; 110 men ). All groups were matched to each other in terms of sex and age.

One test dataset was used for clinical validation, which did not overlap with the data used in the training dataset. The test dataset contains a total of 79 subjects with 237 photographs. There were 14 non-acromegaly people with 43 photographs scored 1 (6 women; 8 men), 31 patients with 93 photographs scored 2 (15 women; 16 men), and 34 patients with 101 photographs scored 3 (17 women; 17 men). All groups were matched to each other in terms of sex and age.

**2.2 Relationship Between Score and Disease Severity**

**2.2.1 P-value**

From the P-value table, we could conclude that our score has a strong Spearman correlation with Tumor size, Tumor maximum diameter, Serum GH level, Serum insulin-like growth factor (IGF)-1 level, and Ki67%.

**2.2.2 Correlation Coefficient**

We used the Spearman correlation coefficient to measure the rank correlation (**Table S1**).

We plotted the heatmap to display the Spearman Correlation coefficient of the score and other features (**Figure S1**) clearly. It was observed that our score had significant monotonicity in terms of tumor size, maximum tumor diameter, serum GH level, serum IGF-1 level, and Ki67%.

**2.3 Evaluation Metrics** **upon Test Dataset**

We used three evaluation metrics, including accuracy, precision, and recall. The confusion matrix was given in **Table S2**. There were 43 score-1, 93 score-2, 101 score-3 photographs. The total prediction accuracy of our proposed model was 90.7%, where 22 photographs had the incorrectly predicted scores. For score-1 class, our model had a precision of 94.1%, a recall of 74.4%, and a F1-Measure of 0.831.

**2.4 Performance Evaluation** **Compared with Doctors**

Ten junior internal medicine physicians had a test upon the test dataset. The accuracy, precision, and recall were shown in **Table S3**. The total prediction accuracy of the physicians was 89.0%. For score-1 class, the physicians had a precision of 91.7%, a recall of 76.7%, and a F1-Measure of 0.835. From **Table S2** and **Table S3** we could see that the total prediction accuracy of our developed computer algorithm was higher than that of our ten junior internal medicine physicians (90.7% vs. 89.0%).

**3 Limitations**

**3.1** Though the restrictions shooting angles were relaxed to within plus or minus 45 degrees centered on the standard coronal plane, overall, we only enrolled the frontal views of acromegaly faces and non-acromegaly faces. If side views could also be incorporated into the training process, a higher accuracy should be expected.

**3.2** Since only a few constraints were placed for taking the face-pictures (e.g., camera model, definition, shooting angle, and image resolution.), there was a potential of systematic error which might exaggerate the true differences and the detection-accuracy. However, since the training data set is huge, we deem that the bias induced by this factor is likely to be small.

**3.3** The data size is still not big enough when compared with Esteva et al.’s work on skin cancer classification by CNN (129,450 clinical images) [6] and Gulshan et al.’s work on diabetic retinopathy detection with deep learning algorithm (128,175 retinal images) [7].

**3.4** Another fundamental limitation arises from the nature of deep learning is that the deep learning system is only imputed with facial photographs without explicit definitions of features (e.g., skeletal structure details). Since the deep learning “learned” the landmarks that are most predictive for the preferability implicitly, it seems that the deep learning uses the characteristics that are ignored by humans or unknown previously to doctors.

**3.5** Even though the face detection is conducted by software, in its current state, manual editing is still necessary to obtain the optimal results. Future research will aim at improving the automatization of this method. Additionally, ethical issues should be considered. Since this software can be used as a diagnostic tool, it should be used with the same precautions as others. For instance, individuals who are tested should be informed of the meaning and potential consequences of both positive and negative results, and the real meaning and background of the severity score. It should not be used without the consent of participating individuals. Moreover, the diagnosis or suspicion of acromegaly, whether correct or not, maybe stigmatized by some people. All these issues should be considered when this type of technique is used in the future.

**References**

1. Haahtela T, von Hertzen L, Anto JM, Bai C, Baigenzhin A, Bateman ED, Behera D, Bennoor K, Camargos P, Chavannes N et al: Helsinki by Nature: The Nature Step to Respiratory Health. Clin Transl Allergy 2019, 9:57.

2. Zhang Z, Chen X, Wang B, Hu G, Zuo W, Hancock ER: Face Frontalization Using an Appearance-Flow-Based Convolutional Neural Network. IEEE Trans Image Process 2019, 28(5):2187-2199.

3. Sagonas C, Ververas E, Panagakis Y, Zafeiriou S: Recovering Joint and Individual Components in Facial Data. IEEE Trans Pattern Anal Mach Intell 2018, 40(11):2668-2681.

4. Gilpin W: Cellular Automata as Convolutional Neural Networks. Phys Rev E 2019, 100(3-1):032402.

5. Liu S, Song Y, Zhang M, Zhao J, Yang S, Hou K: An Identity Authentication Method Combining Liveness Detection and Face Recognition. Sensors (Basel) 2019, 19(21).

6. Esteva A, Kuprel B, Novoa RA, Ko J, Swetter SM, Blau HM, Thrun S: Dermatologist-Level Classification of Skin Cancer with Deep Neural Networks. Nature 2017, 542(7639):115-118.

7. Gulshan V, Peng L, Coram M, Stumpe MC, Wu D, Narayanaswamy A, Venugopalan S, Widner K, Madams T, Cuadros J et al: Development and Validation of a Deep Learning Algorithm for Detection of Diabetic Retinopathy in Retinal Fundus Photographs. JAMA 2016, 316(22):2402-2410.

**Figure Legends**

**Figure 1** Examples of data augmentation methods, from left to right, we had the original image, the image with changed brightness, the image changed saturation, the image added Gaussian noise, the image flipped horizontally.

**Figure 2** The architecture of our proposed model, Conv represents the 1∗1 convolutional layer; the GAP, AvgPool, and FC are the global average pooling layer, the average pooling layer, and the fully connected layer, respectively. In this work, the rate of dropout was set to 0.8, the activation function of the convolution layer is ReLU, and there is no activation function in the fully connected layer.

**Supplemental Files**

**Additional File 1** The materials, methods, results and limitations of this study in detail.

**Additional File 2 (Table S1)** Spearman correlation coefficient results and p-values measuring the rank correlation.

**Additional File 3 (Figure S1)** The heatmap displaying the Spearman Correlation coefficient of the score and other features.

**Additional File 4 (Figure S2)** Face detection: the blue box represented the detected bounding box by the Face Recognition library. The red box represented the bounding box after we increased the height.

**Additional File 5 (Table S2)** Confusion Matrix to Evaluate the Accuracy, Precision, and Recall of the Algorithm Model.

**Additional File 6 (Table S3)** Confusion Matrix to Evaluate the Accuracy, Precision, and Recall of Physicians.
