# Supplementary material for: Constructing an automatic diagnosis and severity-classification model for acromegaly using facial photographs by deep learning
Source: J Hematol Oncol. 2020 Jul 3;13:88. doi: 10.1186/s13045-020-00925-y (PMC7333291; doi:10.1186/s13045-020-00925-y)
Supplement: Supplementary file 2 — Additional file 2:. Table S1. Spearman correlation coefficient results and p-values measuring the rank correlation. [file 13045_2020_925_MOESM2_ESM.doc]

| **Table S1** Spearman correlation coefficient results and p-values measuring the rank correlation | | |
| --- | --- | --- |
| **Feature** | **Spearman Correlation Coefficient** | **P Value** |
| Disease course (months) | 0.2177153616015280 | 0.0006321479960268350 |
| Tumor size (mm^3^) | 0.38793103599519300 | 3.77515720082995E-10 |
| Tumor maximum diameter (mm) | 0.3905263827567930 | 2.8156054198373E-10 |
| Serum GH level | 0.6002823841275900 | 3.51538398129176E-25 |
| Serum IGF-1 level | 0.30048063219481100 | 1.83696706093346E-06 |
| Ki 67% | 0.13129349045549600 | 0.04085777397175900 |
