# Supplementary figures and images for: Constructing an automatic diagnosis and severity-classification model for acromegaly using facial photographs by deep learning
Source: J Hematol Oncol. 2020 Jul 3;13:88. doi: 10.1186/s13045-020-00925-y (PMC7333291; doi:10.1186/s13045-020-00925-y)

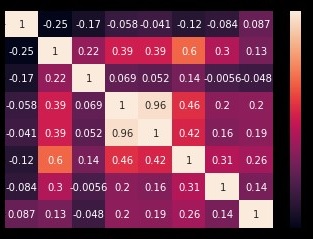

Supplement: Supplementary file 3 — Additional file 3:. Figure S1. The heatmap displaying the Spearman Correlation coefficient of the score and other features. [file 13045_2020_925_MOESM3_ESM.jpg]

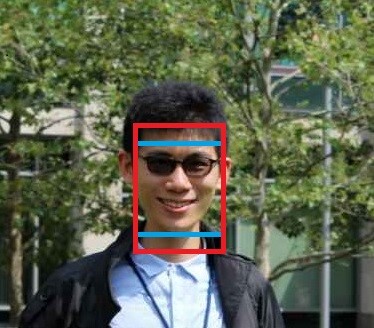

Supplement: Supplementary file 4 — Additional file 4:. Figure S2. Face detection: the blue box represented the detected bounding box by the Face Recognition library. The red box represented the bounding box after we increased the height. [file 13045_2020_925_MOESM4_ESM.jpg]

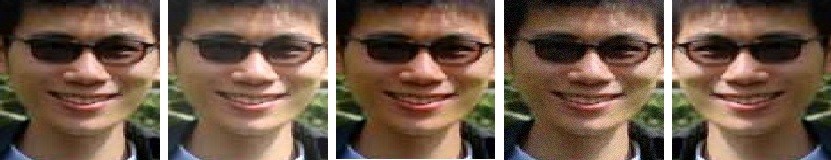

Supplement: Supplementary file 5 — Additional file 5:. Figure S3. Examples of data augmentation methods, from left to right, we had the original image, the image with changed brightness, the image changed saturation, the image added Gaussian noise, the image flipped horizontally. [file 13045_2020_925_MOESM5_ESM.jpg]
